# Solitary Intraosseous Myofibroma of the Mandible in a Nine-Year-Old Child: A Case Report and Literature Review

**DOI:** 10.7759/cureus.64232

**Published:** 2024-07-10

**Authors:** Ahmed Lazim, Samir M Amer, Ghadir M Eltawil, Robert Laski, Riya Kuklani

**Affiliations:** 1 Pathology, Temple University Hospital, Philadelphia, USA; 2 Dentistry, Al Hokail Specialized Digital Polyclinics Academy, Muharraq, BHR; 3 Oral Surgery, Valley Oral Surgery, Allentown, USA

**Keywords:** mandible, oral, pediatric, myofibroma, intraosseous

## Abstract

Myofibroma (MF) is a benign neoplasm derived from myofibroblasts. While they are infrequent, these tumors are predominantly found in the pediatric group and seldom manifest as intraosseous mandibular tumors. Herein, we present a 9-year-old female with a radiolucent lesion in the left mandible associated with malposed left lower canine and 1st premolar teeth. Clinical examination revealed a slightly tender 5×4 cm firm mass resulting in an expansion of the buccal and lingual aspects of the mandible in the canine and first premolar region. An incisional biopsy revealed a benign tumor consisting of spindle cells organized in fascicles, alongside dispersed thin-walled blood vessels. Tumor cells tested positive for α-smooth muscle actin (SMA) and vimentin. Given these findings, a diagnosis of MF was established. To the best of our knowledge, only 45 cases of solitary MF of the mandible have been reported in the pediatric age group in the literature. We describe one additional case and provide a review of the literature.

## Introduction

Spindle cell lesions in the head and neck region are clinically and biologically diverse. Although some of these lesions are malignant, others are benign or simply reactive. Myofibroma (MF), a rare benign spindle cell tumor derived from myofibroblasts, is frequently seen in the head and neck area, predominately in the skin and subcutaneous tissue [1,2]. However, it can also be seen in internal organs such as the lungs, heart, CNS, and pancreas [2]. While they are infrequent, these tumors are the predominant fibrous tumor found in children and seldom manifest as intraosseous mandibular neoplasm [3].

Based on the WHO classification of soft tissue tumors, MF is more suitable for describing individual lesions and falls under the benign category of pericytic (perivascular) tumors due to several shared morphologic features [4]. Because of its rarity, along with vague clinical, radiographic, and morphologic features, there is a potential for MF to be misdiagnosed as benign mesenchymal or low malignant potential tumors [5,6].

To our knowledge, 45 cases of individual intraosseous MF of the mandible in children have been reported in the literature. In this study, we present an additional case of intraosseous MF of the mandible in a 9-year-old female and provide a comprehensive review of the literature.

## Case presentation

**Figure 1:**
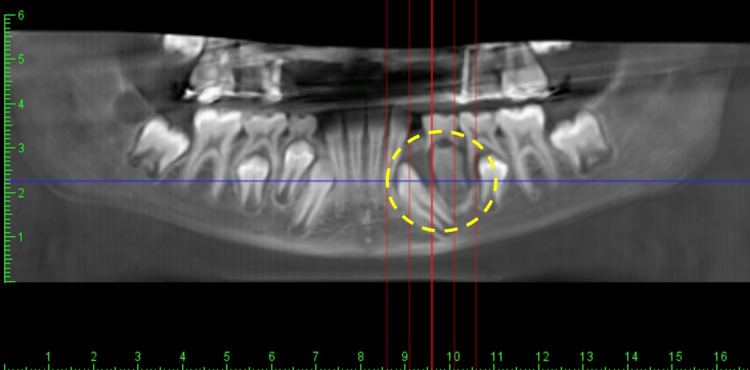
X-ray of the mandible demonstrating a radiolucent ill-defined lesion at the apex of the left lower canine and 1st premolar tooth, marked by a dashed yellow circle.

We present a 9-year-old female referred to an oral surgeon for assessment of a radiolucent lesion of her left mandible associated with malposed left lower canine and 1st premolar teeth, that had slightly increased in size during the past two weeks (Figure 1). She has a past medical history of asthma. Upon clinical examination, a slightly tender, firm mass measuring 5×4 cm was observed, causing expansion of the buccal and lingual aspects of the mandible, particularly in the canine and first premolar region.

**Figure 2:**
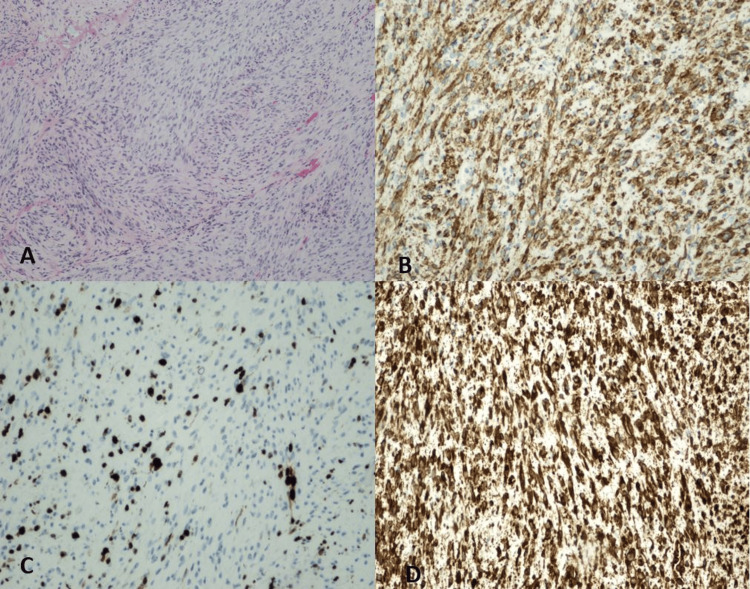
Histological examination. A) Classical morphology of myofibroma showing bland-looking spindle-shaped cells arranged in fascicles with dispersed thin-walled blood vessels (Hematoxylin & Eosin stain, 200×). B,D) The tumor cells show positive staining for SMA and vimentin, respectively. C) Tumor cells staining for Ki67 is low (<5%). SMA: smooth muscle actin

A dental cone-beam computed tomography of the mandible revealed unilocular radiolucency associated with malposed left lower canine and 1st premolar teeth. There were no observed signs of calcifications within the radiolucent lesion. The patient subsequently underwent an incisional biopsy of the lesion. Histological examination revealed a benign tumor consisting of bland spindle-shaped fibroblasts arranged in fascicles with dispersed thin-walled blood vessels (Figure 2A). No necrosis was observed, and mitotic figures were rare. Immunohistochemistry revealed that the tumor cells were positive for α-smooth muscle actin (SMA) and vimentin, and negative for S100, CD34, and desmin. The Ki-67 or mitotic index was interpreted to be low (< 5%) (Figures 2B-2D). Given the histopathological and immunostaining features, a diagnosis of intraosseous MF in the mandible was reached. The lesion was easily separated from the surrounding bone. No immediate reconstruction was performed. At the 12-month follow-up, there was no clinical evidence of tumor recurrence. The patient was unavailable for subsequent follow-up.

## Discussion

MF (solitary) and myofibromatosis (multifocal/multicentric) are a rare benign mesenchymal tumor of fibroblastic origin with perivascular myoid features which are most commonly seen in infancy and childhood [7-9]. In 1951, Williams and Schrum initially identified MFs in a newborn infant and referred to them as congenital fibrosarcoma [10]. Then, in 1954, MFs were described by Stout as “congenital generalized fibromatosis” [11].

MFs are frequently seen in the head and neck area, as well as trunk and extremities, and seldom present as intraosseous neoplasm [1,3]. Mandibular involvement is exceptionally rare. MF can also arise in the internal organs such as muscle, CNS, pancreas, lung, and heart with the soft tissue being the most common site for occurrence [2].

MF of the mandible is a tumor typically observed in children, with a mean age of 7.2 years. It commonly occurs in the first decade of life, showing a clear male predominance with a male-to-female ratio of 2.3:1 [12]. To the best of our knowledge, only 46 cases of solitary mandibular MF have been reported in children in the literature, including our case (Table 1) [3,6,12-31].

**Table 1:** Demographic data, clinical and radiologic presentation, and treatment modality in 46 pediatric patients with mandibular myofibroma. F: female; M: Male; wk: week; mo: month; y: year; NI: No information, CP/RP: clinical and radiologic presentation

Case	Author	Year	Sex	Age	Size (cm)	Duration	CP/RP	Treatment
1	Lazim et al.	Current case	F	9 y	5	2 wk	Unilocular radiolucency associated with malposed left lower canine and 1st premolar teeth	Excision
2	Santos et al. [12]	2023	F	15 y	NI	NI	Hypodense lesion with lobulated limits, as well as expansion and thinning of the cortical bone in the left mandible.	Enucleation and curettage of the lesion
3	Aryanpour et al. [13]	2022	F	4 y	3.6	NI	Large expansile lytic mass involving the right mandibular ramus	Complete resection
4	Hegde et al. [14]	2021	M	7 mo	3	1 mo	Diffuse swelling on the left lower third of the face	Surgical curettage
5	Montalli et al. [15]	2020	M	2 y	3	NI	Localized, firm nonmobile swelling in the inferior aspect of the left side of the body of the mandible	NI
6	Mesquita et al. [16]	2020	F	4 y	NI	6 mo	Swelling in the left mandibular angle associated with pain	Surgical excision
7	Dhupar et al. [17]	2017	F	9 y	2	4 mo	Swelling of the left side of her lower jaw	Enucleation and curettage of the lesion
8	Chattaraj et al. [18]	2017	M	6 y	2	2 mo	Localized swelling in the left body of the mandible	Surgical excision
9	Chinta et al. [19]	2016	M	4 y	3	2 wk	Swelling in the lingual surface of the mandibular anterior	Surgical excision of the tumor en bloc along with a wedge of the mandible
10	Lopes et al. [20]	2015	F	2 y	5	NI	Slight mandibular swelling	Surgical excision
11	Rai et al. [21]	2014	F	5 y	2	3 mo	Painless swelling	Surgical excision
12	Urs et al. [22]	2014	M	5 y	4.5	6 mo	Swelling of right side of the mandible	Surgical resection
13	Sundaravel et al. [23]	2013	M	16 y	2	2 mo	Nontender swelling in the right buccal aspect of the mandible	Local-wide surgical excision of the lesion
14	Haspel et al. [24]	2012	F	6 wk	3	3 wk	An intraoral swelling in the left side of her mandible.	Surgical excision
15	Abramowicz et al. [25]	2012	NI	2-12 y	NI	NI	NI	Enucleation and curettage
16	Abramowicz et al. [25]	2012	NI	2-12 y	NI	NI	NI	Enucleation and curettage
17	Abramowicz et al. [25]	2012	NI	2-12 y	NI	NI	NI	Enucleation and curettage
18	Abramowicz et al. [25]	2012	NI	2-12 y	NI	NI	NI	Enucleation and curettage
19	Abramowicz et al. [25]	2012	NI	2-12 y	NI	NI	NI	Enucleation and curettage
20	Jagannathan et al. [26]	2011	F	2 y	5	3 mo	Rapidly increasing extra-oral swelling over the left lower jaw	Surgical excision
21	Nouri et al. [27]	2011	M	16 y	NI	NI	NI	NI
22	Nirvikalpa and Narayanan[28]	2011	M	10 mo	3	5 mo	Painless swelling in the mandibular left posterior region	surgical excision
23	Souza et al. [6]	2009	F	7 y	2	NI	Radiolucent lesion at the base of the right mandible	Excisional biopsy
24	Ech-Charif et al. [29]	2008	NI	18 mo	NI	NI	NI	NI
25	Shibuya et al. [30]	2008	M	12 y	NI	NI	Globe like radiolucent lesion in the left angle of the mandible	NI
26	Allon et al. [31]	2007	F	5 mo	3	1 mo	Asymptomatic swelling	Excision
27	Allon et al. [31]	2007	F	7 y	2	Months	Asymptomatic swelling	Excision
28	Allon et al. [31]	2007	M	4.5 y	5	2 y	Limited mouth opening	Resection
29	Allon et al. [31]	2007	M	4.5 y	3	3 wk	Asymptomatic swelling	Excision
30	Odell et al. [31]	2004	M	10 y	NI	4-6 wk	Rapidly growing intraoral soft tissue mass	NI
31	Troulis et al. [31]	2004	M	6.5 y	NI	NI	NI	Resection and bone graft
32	Montgomery et al. [3]	2000	M	9 mo	NI	NI	NI	NI
33	Montgomery et al. [3]	2000	M	1 y	0.7	NI	NI	NI
34	Lingen et al. [31]	1995	F	NI	NI	1-6 mo	NI	Excision
35	Sugatami et al. [31]	1995	M	2 mo	NI	NI	Diffuse swelling involving the anterior mandible	Excision
36	Jones et al. [31]	1994	M	5 mo	3	2 wk	NI	NI
37	Jones et al. [31]	1994	M	8 y	1.5	NI	NI	NI
38	Jones et al. [31]	1994	M	14 y	3	NI	NI	NI
39	Vigneswaran et al. [31]	1992	F	2 y	2	NI	Buccal expansion	Excision
40	Vigneswaran et al. [31]	1992	F	11 y	2	NI	Radiolucent lesion	Excision
41	Vigneswaran et al. [31]	1992	F	6 y	2	2 wk	Painless localized mandibular swelling	Excision
42	Inwards et al. [31]	1991	NI	6 mo-6 y	NI	NI	NI	Excision
43	Inwards et al. [31]	1991	NI	6 mo-6 y	NI	NI	NI	Excision
44	Inwards et al. [31]	1991	NI	6 mo-6 y	NI	NI	NI	Resection
45	Matthews et al. [31]	1990	M	6 y	2.5	NI	Mass in the right posterior mandible	Excision
46	Slootweg and Muller [31]	1984	M	Newborn	2	NI	Swelling of the anterior part of the left mandible	Excision

When the tumor arises in bone, it expands through infiltration, potentially causing cortical expansion, displacement of the tooth, or root resorption [28]. In our case, the patient presented with a firm mass causing expansion of both buccal and lingual aspects of the mandible in the left canine region, accompanied by malpositioned left lower canine and 1st premolar teeth.

The genetic origin of the MF/myofibromatosis is still unclear, with reports suggesting both autosomal dominant and autosomal recessive inheritance. Mutations in PDGFRB, NOTCH3, and PTPRG genes have been linked to autosomal dominant infantile myofibromatosis [32].

Radiologically, MF typically manifests as unilocular or multilocular radiolucent lesions with well-demarcated borders. In our case, we observed a unilocular osteolytic lesion displacing the left lower canine and 1st premolar teeth. A recent study conducted by Allon et al found that, in a radiological review of intraosseous mandibular MF involving 17 cases, 70% exhibited unilocular lesions, while 30% displayed multilocular lesions. Additionally, 67% of all cases were characterized by well-defined borders [31]. Based on these radiological findings, there is a potential for MF to be mistakenly diagnosed with other benign or low-grade tumors such as ameloblastoma, ameloblastic fibroma, and odontogenic keratocyst [6].

Histologically, MF typically exhibits a nodular biphasic growth pattern architecture with a less cellular peripheral zone featuring short fascicles and bundles of spindle cells with elongated vesicular nuclei, indistinct cell borders, and eosinophilic cytoplasm [3]. In contrast, the central zone is relatively more cellular and consists of round or spindle cells with dark-stained nuclei and scant cytoplasm, associated with thin-walled ectatic hemangiopericytic vessels [2].

Immunohistochemistry plays a pivotal role in distinguishing MF from benign and malignant lesions. In MF, the spindle cells typically exhibit positive staining for SMA and vimentin [14]. The absence of staining for S100 (a marker for tumors of nerve cell origin), CD34 (a marker for vascular tumors), and desmin (a marker for malignant smooth muscle and fibroblastic tumors) in the current case supports the diagnosis of MF and rules out the possibility of neuroma, hemangiopericytoma, leiomyosarcoma, and fibrosarcoma, respectively [14].

The preferred treatment for individual MFs is conservative surgical excision [26]. In cases where the lesion is extensive and causes significant destruction, aggressive segmented jaw resection may be considered [33]. Given that MFs in the mandible often affect pediatric patients, additional reconstructive surgery is commonly necessary. Recurrence following surgical removal is rare, typically occurring only in cases with anatomical complexity that make complete tumor removal challenging [33].

## Conclusions

Mandibular intraosseous MF is extremely rare in the pediatric population. Achieving an accurate diagnosis requires a comprehensive assessment, including clinicopathologic correlation, evaluation of radiological and immunohistochemical features, and at times, molecular studies. This is crucial for distinguishing MF from other benign soft tissue neoplasms that may share morphological and radiographic similarities. Despite its rarity, MF should be included in the differential diagnosis when assessing unilocular or multilocular radiolucent lesions of the jaw.
